# Mandibular Hemangiomatous Ameloblastoma: A Case Report and Literature Review

**DOI:** 10.1155/crid/7584900

**Published:** 2026-07-21

**Authors:** S. Shanmuha Priya, Vinayakrishna Kolari, Soniya Adyanthaya, Shakil Moidin, K. M. Raina

**Affiliations:** ^1^ Department of Oral Pathology and Microbiology, Yenepoya Dental College, Yenepoya (Deemed To Be University), Mangalore, Karnataka, India, yenepoya.edu.in; ^2^ Department of Oral and Maxillofacial Surgery, Yenepoya Dental College, Yenepoya (Deemed To Be University), Mangalore, Karnataka, India, yenepoya.edu.in

**Keywords:** ameloblastoma, hemangiomatous, plexiform type, tumourigenesis, vascular component

## Abstract

Hemangiomatous ameloblastoma is an exceedingly rare histologic variant of ameloblastoma, characterized by extensive vascularity. The present case discusses the hemangiomatous ameloblastoma in a 60‐year‐old male patient. The patient presented with a swelling in the mandibular anterior region that persisted for 2 years. Radiographic examination revealed a unilocular, well‐defined hypodense lesion with scalloped borders and multilocular areas extending from the mandibular left first molar to the right second molar. Based on these findings, a primary provisional diagnosis of aggressive odontogenic tumour was made. An incisional biopsy was taken and the lesion was diagnosed as ameloblastoma. Following this, hemimandibulectomy was performed and the specimen was sent for histopathological examination. Microscopic examination demonstrated odontogenic epithelial islands with peripheral columnar ameloblast‐like cells, stellate reticulum‐like cells replaced by proliferating round cells, and extensive endothelial‐lined vascular channels filled with red blood cells. The lesion showed plexiform patterns, dentinoid‐like material and areas of calcification. The final diagnosis was given as hemangiomatous ameloblastoma. Hemangiomatous ameloblastoma differs from conventional ameloblastomas in its vascular components, with proposed etiopathogenetic mechanisms including angiogenesis, hamartomatous malformation, or epigenetic VEGF expression. Despite its rarity and vascularity, treatment follows conventional ameloblastoma protocols. Surgical management poses challenges due to its potential profuse bleeding. Although its biological behaviour and recurrence rates are not fully understood, this case highlights the importance of including vascular lesions in differential diagnoses and highlights the need for further studies to elucidate its etiopathogenesis and prognosis.

## 1. Introduction

Ameloblastoma is a slow‐growing cystic or solid benign locally aggressive odontogenic neoplasm of the jaws. They have a variety of histologic pattern which represent their polymorphic nature. The two main histologic subtypes are follicular and plexiform followed by acanthomatous, granular cell, desmoplastic and basal cell types. Keratoameloblastoma, palliferous keratoameloblastoma, unicystic ameloblastoma, desmoplastic ameloblastoma and unicystic ameloblastoma are the less common variants that are encountered [1]. Despite the different histomorphologic patterns, there are no significant differences in the biologic behaviour and prognosis of ameloblastoma. The possible exceptions could be unicystic and desmoplastic ameloblatoma [2].

Hemangiomatous ameloblastoma is a rare histologic variant which was originally described as ameloblastoma in which portion of the tumour contains spaces that are filled with blood and endothelial lined capillaries. Hemangiomatous ameloblastoma differs in their histologic and radiological features from the conventional ameloblastoma. Origin of this variant is not completely resolved. Although rare and highly vascular, treatment follows standard ameloblastoma protocols, with no reported recurrence in cases monitored for up to 2 years. Surgical management remains challenging due to the risk of significant bleeding. Although its biological behaviour and recurrence patterns are not yet fully understood, the present case emphasizes the importance of considering vascular lesions in differential diagnoses and highlights the need for further research to clarify its etiopathogenesis and prognosis [3].To our best knowledge, only 25 cases have been reported in the literature including the present case.

A rare case of hemangiomatous ameloblastoma of the mandibular anterior region occurring in a 60‐year‐old male patient is discussed here.

## 2. Case Description

Written informed consent was obtained from the patient for the publication of this case and images

**Figure 1:**
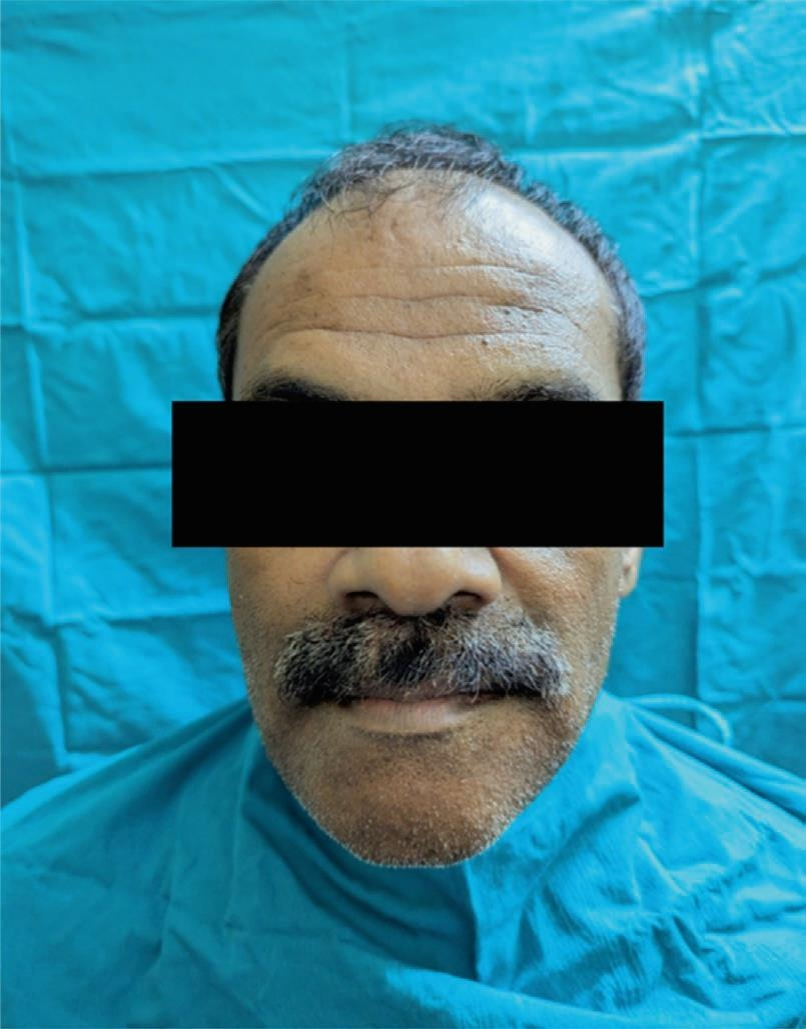
Extraoral image showing swelling of mandible with facial asymmetry.

**Figure 2:**
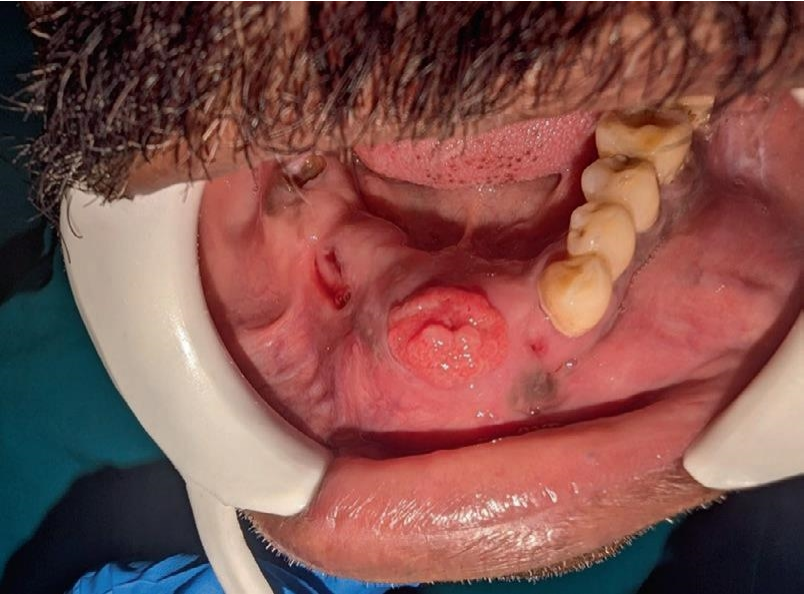
Intraoral image showing growth in mandibular anterior region.

**Figure 3:**
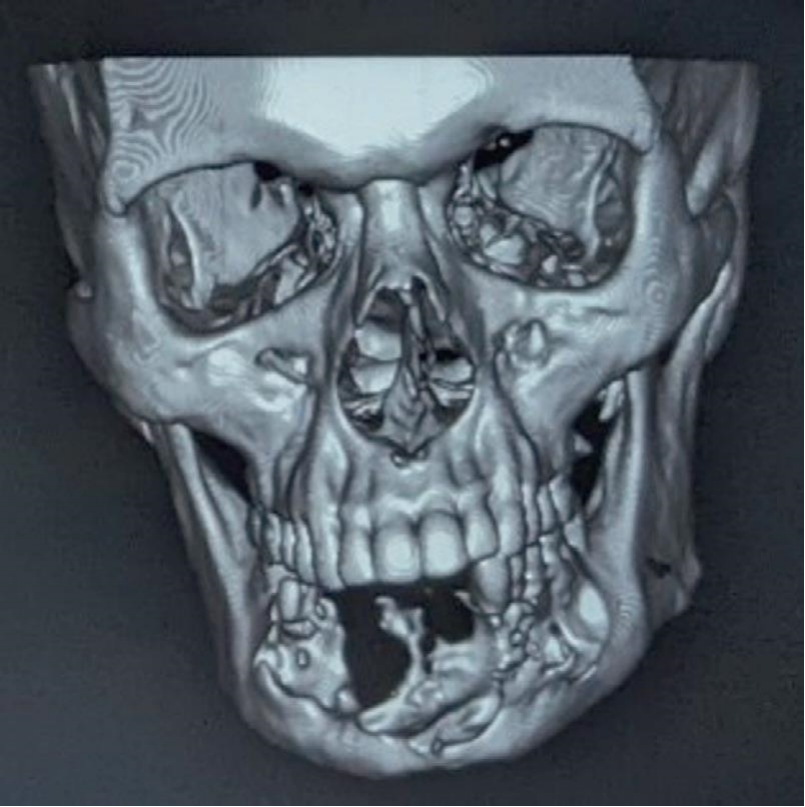
CBCT shows a unilocular well‐defined expansile lesion with a multilocular region.

A 60‐year‐old male patient reported to the Department of Oral Medicine and Radiology with a chief complaint of swelling in the lower anterior jaw region for the past 2 years. Extraoral examination revealed a diffuse, solitary swelling measuring approximately 10 × 4 cm, extending anteroposteriorly from the right body of the mandible to the left body (Figure 1). Intraoral examination showed a solitary growth of about 2 × 2 cm in the mandibular anterior region (Figure 2). A cone beam computed tomography (CBCT) revealed a unilocular, well‐defined, expansile hypodense lesion with a scalloped border, along with a multilocular region, extending from the left mandibular first molar to the right mandibular second molar (Figure 3). Based on these clinical and radiographic features, an aggressive odontogenic tumour was considered the primary differential diagnosis. An incisional biopsy was done and the provisional diagnosis of ameloblastoma was given. Following which hemimandibulectomy was performed. The excised specimen was firm, reddish‐brown and glistening in appearance and sent for histopathological examination.

**Figure 4:**
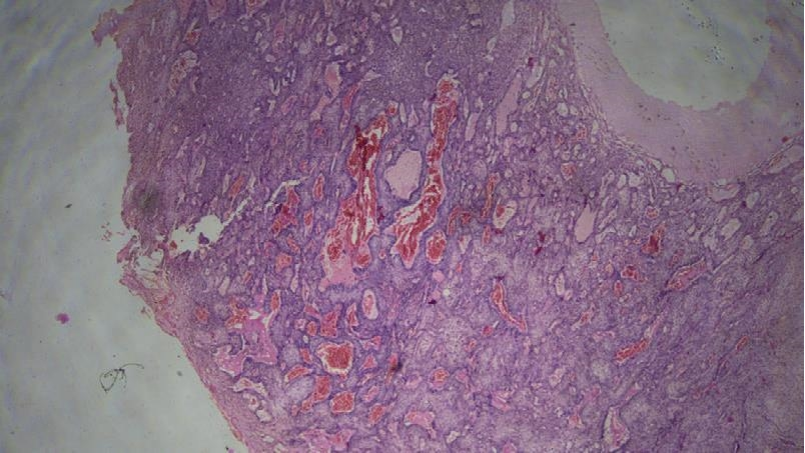
Odontogenic epithelial islands with extensive vascular components in 4x.

**Figure 5:**
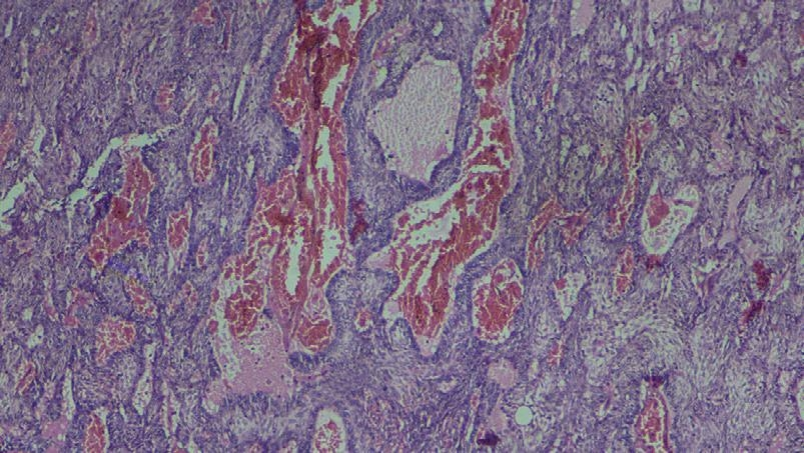
Extensive vascular components with large blood‐filled spaces and numerous endothelial‐lined channels in 10x.

Microscopic examination revealed odontogenic epithelial islands exhibiting peripheral tall columnar ameloblast‐like cells and central stellate reticulum‐like cells. In many areas, stellate reticulum‐like cells were replaced by highly proliferating round to ovoid cells. Extensive vascularity with numerous endothelial‐lined channels and large blood‐filled spaces were evident (Figures 4 and 5). Columnar cells arranged in anastomosing strands with inconspicuous stellate reticulum‐like cells exhibiting a plexiform pattern were observed. Areas showing inductive changes with dentinoid‐like material and areas of calcification were also observed. In the areas of cystic degeneration, ameloblastomatous epithelium lining the cystic cavity was noted. Correlating with the clinical, radiographical and histopathological features, the tumour was diagnosed as hemangiomatous ameloblastoma.

## 3. Discussion

Ameloblastoma is one of the most common odontogenic tumours. Ameloblastoma occurs due to faulty differentiation of enamel organ to a point of actual enamel formation [4].

Hemangiomatous ameloblastoma is an extremely rare variant of ameloblastoma that exhibits extensive vascularity, histologically present as odontogenic epithelial islands with extensive vascular components containing numerous endothelial lined blood vessels. Kuhn in 1932 described the first case of hemangiomatous ameloblastoma as a combination of hemangioma and adamantinoma. A similar lesion was reported by Aisenberg in 1950 as adamantinohemangioma. Between 1957 and 1971 several case reports were published describing this entity under various terminologies such as ameloblastic hemangioma, adamantine‐hemangioma, hemangio‐ameloblastoma, vascular ameloblastoma and ameloblastoma with vascular component [5]. The next case was reported in 2001 after a gap of 30 years [6].

A literature search was performed using PubMed/Google Scholar using the keyword hemangiomatous ameloblastoma. A total of 24 cases were published in the English literature from 1950 to 2024. Table 1 summarizes the literature review of published cases including the present case.

**Table 1:** Review of literature from 1950 to 2024.

Author/year	Age/sex	Location	Radiographic features	Histopathological features	Follow‐up
Aisenberg/1950 [7]	48/F	Mandible	Bone destruction	Endothelial lined capillaries with blood in cystic spaces	2 years with no recurrence
Lucas et al./1952 [7]	45/F	Mandible	Typical ameloblastoma	Dilated capillaries and blood in cystic spaces in plexiform and follicular pattern	NA
Villa et al./1953 [7]	—	Mandible	—	Coalescing capillaries and blood filled cavities in a plexiform pattern	NA
Oliver et al./1961 [7]	33/F	Mandible	Radiolucency	Dialted vascular channel in simple ameloblastoma	2 years with no recurrence
Shklar et al./1965 [7]	13/M	Mandible	Well defined radiolucency	Endothelial lined vascular channels in a plexiform pattern	NA
Gardner et al./1966 [7]	76/F	Mandible	Radiolucency	Dilated capillaries blood filled cavities in a plexiform pattern	NA
Grover et al./1971 [7]	46/M	Mandible	Radiolucency	Endothelial lined lumens formed blood elements in ameloblastoma without a stellate reticulum	No comlication after 4 weeks
Van Rensberg et al./2001 [7]	26/F	Mandible	Well defined mixed radioopacity/radiolucency	Endothelial lined channels, blood filled spaces, thrombus formation in a plexiform ameloblastoma	NA
Tamgadge et al./2010 [7]	31/M	Mandible	Well defined radiolucency	Endothelial lined channels and blood filled spaces in plexiform pattern	Good healing was seen after 4 months
Jois et al./2012 [7]	42/M	Mandible	Poorly defined mixed radiolucency/radioopacity	Cavernous endothelial lined channels in plexiform pattern	No evidence of recurrence of 2 years
Sharma et al./2012 [7]	15/M	Maxilla	Well defined radiolucency	Endothelial lined channels and blood filled spaces in follicular pattern	NA
Sharode et al./2013 [7]	18/M	Mandible	Multilocular radiolucency	Endothelial lined blood vessels in unicystic ameloblastoma	NA
Maheshwari et al./2013 [7]	51/M	Mandible	Radiolucency	Giant cells and vascular spaces in unicystic ameloblastoma	NA
Rajamohan et al./2013 [7]	20/M	Mandible	Multilocular radiolucency	Vascular spaces in a plexiform pattern	NA
Hegde et al./2015 [7]	18/F	Mandible	Multilocular radiolucency	Dilated endothelial blood vessels in a plexiform pattern	18 months
Kasangari et al./2015 [7]	24/F	Mandible	Multilocular radiolucency	Endothelial lined blood vessels with extravasated blood elements in unicystic pattern	1 year
Venigalla et al./2018 [7]	35/F	Mandible	Mixed radiolucency radioopacity	Endothelial lined blood vessels in desmoplastic and plexiform pattern	15 months
Childers EL et al./2020 [7]	64/F	Mandible	Unilocular radiolucency	Endothelial lined blood vessels in solid microcystic pattern	2 years
Saxena et al./2020 [8]	32/M	Maxilla	Mixed radiolucency	Blood filled sinus spaces in a follicular pattern	NA
Sachdev et al./2022 [9]	43/F	Maxilla	Multilocular radiolucency	Endothelial lined blood elements with extravasated blood in a plexiform pattern	NA
Puri et al./2022 [7]	38/M	Mandible	Multilocular mixed radiolucency	Dilated endothelial lined blood vessels with extravasated RBC′s in follicular and plexiform pattern	NA
Patil et al./2023 [10]	22/M	Maxilla	Unilocular radiolucency	Prominent vascular component in a intramural plexiform pattern	No evidence of recurrence after 2 years follow‐up
Gupta et al./2023 [11]	16/F	Mandible	Multilocular radiolucency	Presence of large blood filled spaces with numerous endothelial lined capillaries in a plexiform pattern	NA
Trimukhe et al./2024 [12]	33/M	Mandible	Multilocular radiolucency	Hemorrhage and endothelial lined vessels engorged with RBC′s in plexiform and follicular pattern	Under follow‐up
Present	60/M	Mandible	Multilocular radiolucency	Extensive vascular components with numerous endothelial lined channels and large blood filled spaces in a plexiform pattern	Under follow‐up

Abbreviation: NA, not available.

The mean age of occurrence of hemangiomatous ameloblastoma is about 35.4 years with the range from 13 to 76 years. Maxillary involvement was identified in three cases and mandibular involvement was seen in 21 cases. Males and females were equally affected, with an M:F ratio of approximately 1:1. In the present case hemangiomatous ameloblastoma occurred in the mandibular anterior region in a 60‐year‐old male patient. Hemangiomatous ameloblastoma shows a variable radiographic picture ranging from mixed to multilocular radiolucency [13, 14]. Microscopically, hemangiomatous ameloblastoma is composed of odontogenic epithelial islands with ameloblast‐like cells and extensive vascular component in odontogenic islands and stroma. The most common pattern seen in association with hemangiomatous ameloblastoma is the plexiform type. The presence of extensive vascular component in ameloblastoma is not fully understood [15, 16]. Various theories have been proposed to explain the vascular components of hemangiomatous ameloblastoma. These include separate neoplasm, angiogenesis during tumour development, hamartomatous malformation, secondary change, traumatic incident, collision tumour and degenerative process. The other possible mechanism for tumorigenesis in hemangiomatous ameloblastoma is the excessive expression of vascular endothelial growth factor by an epigenetic mechanism. This vascular component of hemangiomatous ameloblastoma did not seem to have any effect on recurrence [17, 18].

Histopathological differential diagnosis includes hemangioma, aneurysmal bone cyst, angiosarcoma, telangiectatic osteosarcoma, angiomatoid malignant fibrous histiocytoma. Histopathological differential diagnosis with key differentiating feature is given in Table 2.

**Table 2:** Histopathological differential diagnosis with key differentiating features.

Hemangioma	Numerous large dilated vascular spaces lined by endothelial cells
Aneurysmal bone cyst	Numerous empty empty or blood filled sinusoidal spaces separated by fibrous stroma containing multinucleated giant cells
Angiosarcoma	Multiple bizarre endothelial cells with numerous anastomosing vascular channels and necrotic changes
Telangiectatic osteosarcoma	Empty or blood filled cystic spaces with hpercellular anaplastic stromal cells with minimal osteoid
Angiomatoid malignant fibrous histiocytoma	Fibrohistocytic tumour characteristics with vascular component

Hemangiomatous ameloblastoma is treated similar to that of the conventional ameloblastoma. High vascular nature of this lesion may pose an intraoperative challenge to the surgeon [19, 20]. Previous reports showed no recurrences or complications after varied periods of follow‐up. In approximately 5 cases, no recurrence was observed during the 2 years follow‐up period. In the present case, the patient is under follow‐up with no evidence of recurrence.

## 4. Conclusion

The biological behaviour of hemangiomatous ameloblastoma is not completely understood because only very few cases are reported in the literature. Vascular lesions should be included in the differential diagnosis of hemangiomatous ameloblastoma. Furthermore, additional studies have to be carried out to understand the exact biological behaviour and etiopathogenesis of this tumour.

## Funding

No funding was received for this manuscript.

## Disclosure

All authors have read and approved the final version of the manuscript.

## Conflicts of Interest

The authors declare no conflicts of interest.

## Data Availability

Data sharing is not applicable to this article as no datasets were generated or analysed during the current study.
